# Data-driven method for damage localization on soft robotic grippers based on motion dynamics

**DOI:** 10.3389/frobt.2022.1016883

**Published:** 2022-11-28

**Authors:** Arsen Abdulali, Seppe Terryn, Bram Vanderborght, Fumiya Iida

**Affiliations:** ^1^ Engineering Department, Cambridge, United Kingdom; ^2^ Robotics and Multibody Mechanics (R&MM), Vrije Universiteit Brussel (VUB), Flanders Make, Brussels, Belgium; ^3^ Physical Chemistry and Polymer Science (FYSC), VUB, Brussels, Belgium

**Keywords:** damage detection, damage localization, data-driven modeling, LSTM, soft gripper

## Abstract

Damage detection is one of the critical challenges in operating soft robots in an industrial setting. In repetitive tasks, even a small cut or fatigue can propagate to large damage ceasing the complete operation process. Although research has shown that damage detection can be performed through an embedded sensor network, this approach leads to complicated sensorized systems with additional wiring and equipment, made using complex fabrication processes and often compromising the flexibility of the soft robotic body. Alternatively, in this paper, we proposed a non-invasive approach for damage detection and localization on soft grippers. The essential idea is to track changes in non-linear dynamics of a gripper due to possible damage, where minor changes in material and morphology lead to large differences in the force and torque feedback over time. To test this concept, we developed a classification model based on a bidirectional long short-time memory (biLSTM) network that discovers patterns of dynamics changes in force and torque signals measured at the mounting point. To evaluate this model, we employed a two-fingered Fin Ray gripper and collected data for 43 damage configurations. The experimental results show nearly perfect damage detection accuracy and 97% of its localization. We have also tested the effect of the gripper orientation and the length of time-series data. By shaking the gripper with an optimal roll angle, the localization accuracy can exceed 95% and increase further with additional gripper orientations. The results also show that two periods of the gripper oscillation, i.e., roughly 50 data points, are enough to achieve a reasonable level of damage localization.

## 1 Introduction

Soft robotics is an emerging field that complements traditional robotics with a flexible structure that simplifies interaction with complex environments due to an excessive degree of freedom. This beneficial characteristic of soft robots, however, raises new challenges going far beyond the theory of traditional robotics. One downside of soft robots is that soft matter is prone to damage during contact with sharp objects. Even small damage that keeps the robot functional at early stages tends to propagate during operation and render the robot impractical (Kingsley and Quinn, 2002). Self-healing materials are able to recover from such damage but requires keeping the robot idle for some time (Terryn et al., 2021). Therefore, damage detection and localization are of utmost importance.

The general trend in damage detection follows the bio-inspired approach of nervous tissue that mimics the pain perception of living organisms (Li et al., 2021). To simulate the nervous system of the skin, researchers commonly embed a network of sensors into the soft matter of a robot. For instance, Markvicka et al. (2019) proposed to fill the matrix of elastomer with droplets of liquid metal. Damage in such a system leads to a change in the local conductivity by creating electrically conductive pathways. In the study by Thostenson and Chou (2006) and Hong and Su (2012), the strain and damage of material were measured by embedding carbon nanotube and conductive microwire networks, respectively. Pu et al. (2018) proposed the use of a carbon nanotube network for both detecting cracks and self-healing by increasing local temperature through applied electric current. Similarly, Khatib et al. (2020) embedded the flexible conductive carbon black wire into the layered architecture of the artificial skin. Damage to this network cuts the current flow, which helps to detect the damage. A single conductive carbon black wire was used for damage detection in the study by Georgopoulou et al. (2021) by monitoring the strain signal. In order to localize the damage, George Thuruthel et al. (2021) proposed the use of a network of air chambers connected to a piezoresistive pressure sensor. The sudden changes in pressure indicate the damage. A couple of these sensors allow localizing the damage by estimating the delay between spikes in pressure signals. The main disadvantage of these techniques is that the sensors are embedded in the manufacturing process and are difficult to apply to a readily available soft robot. Additionally, these methods can be more prone to failure in high-demand repetitive usage due to additional sensor circuits and wiring, which makes this approach less favorable for industrial applications, e.g., soft grippers (Hughes et al., 2016). Non-invasive damage detection is also crucial for cost reduction, where damage detection sensors are continuously in use, while the worn-out robotic elements can be replaced multiple times. The ultimate goal of the current research is to detect and localize the damage without embedding the sensing elements into the soft matter.

The non-invasive damage detection and localization have been mainly researched for rigid civil infrastructure like buildings, bridges, and railways. (Salawu, 1997; Avci et al., 2021). One of the typical ways to detect the damage is to induce high-frequency vibrations to the rigid structure and analyze feedback using a network of accelerators (Avci et al., 2021). To detect the damage, the signals can be processed in time (Cunha and Caetano, 2006), frequency (Gul and Catbas, 2008; Padil et al., 2020), and time–frequency (Ghahari et al., 2017; Abazarsa et al., 2016) domains by fitting corresponding parametric models. Statistical approaches that are typically used for modeling time-series processes can also be used for damage analysis. For instance, Krishnan Nair and Kiremidjian (2007) and Carden and Brownjohn (2008) utilized the autoregressive moving average (ARMA) model to extract features from the signals. Similarly, Goi and Kim (2017) employed a multivariate autoregressive (AR) model to detect damage on a truss bridge. The statistical model with exogenous input was presented by Gul and Catbas (2011) and Ay and Wang (2014). Recently, machine and deep-learning approaches have been actively utilized for damage prediction on civil structures (Avci and Abdeljaber, 2016; Li and Zhang, 2022; Khodabandehlou et al., 2019; Choe et al., 2021). For instance, Li et al. applied a physics-informed neural network to detect damage on a wind turbine (Choe et al., 2021). Likewise, a convectional neural network (CNN) was used in the study by Khodabandehlou et al. (2019). Since most of the vibration-based approaches are primarily designed for rigid structures, it is challenging to employ them for soft robotics. Attaching rigid accelerometers to the surface of the soft object might seriously deteriorate its elasticity, which is a paramount material property in soft robotics.

In this paper, we present a novel non-invasive data-driven approach for damage detection and localization on a soft gripper based on its motion dynamics. The essential idea is to utilize the key property of non-linear dynamics, i.e., small variations in the input lead to considerable changes in the output in time. Thus, we hypothesize that the minor changes in morphology or material due to damage can produce noticeable changes in feedback in time that can be used for detection and localization of damage. Additionally, the dynamics of the lifted object depend on the external action, which provides an additional variable that can help to track changes in the dynamics. To test the hypothesis, we developed the classification model based on the bi-directional long short-time memory (biLSTM) network that enables us to discover unobvious patterns from time-series data of dynamics and map them to a finite number of damage combinations. We tested the proposed model under eight action conditions, i.e., shaking the gripper with different roll angles, for various time horizons. The experiment results reveal a high classification accuracy for 43 combinations of damage with 99% detection and over 97% localization rates. The results also confirm the importance of the choice of gripper orientation and the minimum number of timesteps.

The current paper is structured as follows: in Section 2, we introduce the classification model for the detection and localization of damage based on the biLSTM network. The experimental setup and conditions are presented in Section 3. Datasets that we prepared for training and testing in Section 3.1 and Section 3.2 were used in evaluation which is presented in Section 4. The conclusion along with a discussion on future directions is provided in Section 5.

## 2 Damage modeling

**FIGURE 1:**
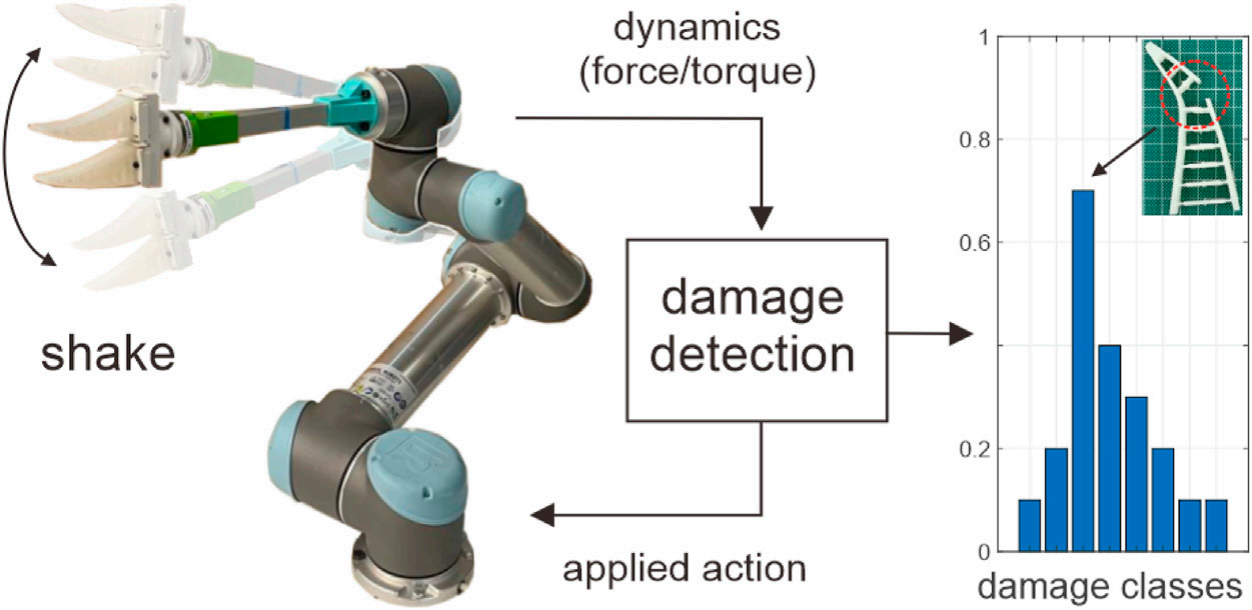
Overview of the proposed damage detection and localization concept. The algorithm applies various action conditions and tracks the change in dynamics (force/torque feedback) in time. Resultant feedback is then used to classify the damage into one of the pre-trained damage configurations.

In this section, we develop a model that identifies and localizes damage on a soft object (Fin Ray gripper in the current case) based on its dynamics at the mounting point. Our ultimate goal is to relate minor changes in dynamics of the object, i.e., force and torque feedback, to the location of the damage (see Figure 1). This problem is generally non-linear, and building an analytical physics-based model is intractable. There are multiple sources of nonlinearity-related morphology, material, and damage conditions. For instance, in case of partial damage, due to asymmetric deformation of the gripper, torque feedback is anisotropic. Furthermore, as the damage occurs in the inner side of the gripper, force feedback is also anisotropic (force patterns in one direction differ from the ones in the opposite direction). In case of complete damage, on the other hand, the self-collision of the hanging-out part of the gripper provides step-like force/torque feedback (contact non-linearity). Additionally, there is no smooth transition, for example, from partial to complete damage, where the feedback profile changes dramatically. Considering the system from the material science point of view, the complex morphology of the gripper fingers and large deformation introduce geometric non-linearity. The visco-elastic nature of the silicone finger introduces a material non-linearity. To leverage this problem, we take a data-driven approach and develop a model based on the bi-directional long short-time memory (biLSTM) neural network. The LSTM is a recurrent neural network (RNN) that was originally introduced by Hochreiter and Schmidhuber (1997) to enable learning a temporal dynamic behavior (Heindel et al., 2022) and time-series data in general (Mayer et al., 2008; Graves et al., 2008; Zia and Zahid, 2019; George Thuruthel et al., 2022). The main advantage of the LSTM is that it partially solves the vanishing gradient problem, which is typically observed in the training of classic RNN. Bi-directional implementation of the LSTM also allows learning both backward and forward information at each step of time (Graves and Schmidhuber, 2005).

We set up the task of damage detection in the current work as a classification problem. Even though the biLSTM network can be utilized for both regression and classification tasks, it is still quite challenging to define the continuous space of the damage with respect to object morphology. Additionally, the regression model requires to populate the input space with the data, which leads to a need of considerably larger set of damaged samples. Taking into account the morphology of the two-fingered Fin Ray gripper, we believe that the classification of the damage into 43 states is sufficient and practical (see Section 3.1 for additional information). The proposed approach should be also applicable to other types of actuators, for e.g., cable-driven soft grippers, where the actuation is needed to be turned off and the model be retrained for individual gripper designs (Chen et al., 2018). The current approach might be less sensitive to punctures in pneumatic grippers, for e.g., PneuNets grippers (Mosadegh et al., 2014). The damage in pneumatic systems, however, can be detected by simply monitoring the pressure in chambers or even localized by using the time-of-arrival difference approach (George Thuruthel et al., 2021), which is not available in cable-driven and passive grippers. Therefore, we believe that the proposed approach closes a significant gap in damage detection and localization in soft grippers.

**FIGURE 2:**
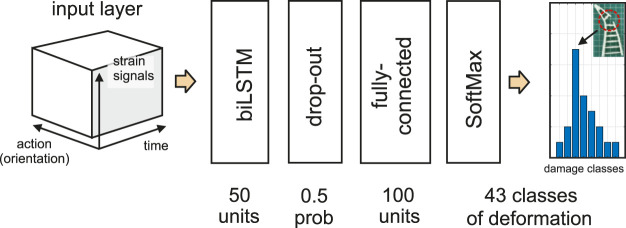
Architecture of the proposed damage detection/localization model.

Another important aspect is the input space of the model. We define the model input as a three-dimensional space (see Figure 2). The first dimension of the model denotes the feedback dimension at the mounting point where we actually measure the dynamics. In our setup, six strain signals correlated to three-dimensional force and torque vectors are sensed at the mounting point of the gripper. The second dimension of the model space describes the applied actions, which in our case is the orientation (roll angle) of the gripper. Other types of actions, for e.g., changing frequency, changing oscillatory patterns, or various motion trajectories, can also be used depending on the equipment specifications and gripper morphology. The roll angle of the gripper, in our case, was sufficient by providing a high level of both damage detection and localization (see Section 4.2 for further analysis). Furthermore, the admittance-type robotic device, which is commonly used in most robotic applications, provides a narrow dynamic range. This makes frequency modulation, for instance, less practical. The last dimension of the model is the time, i.e., the number of data-points in a sequence. Longer time series accommodate a wider range of oscillation frequencies, which in turn helps to discover the changes in dynamics. We assessed the effect of both sizes of action and time space in Section 4.2.

### 2.1 Model architecture

The architecture of the classification model is depicted in Figure 2. We use a single-layer biLSTM network with 50 hidden units. The number of hidden units was empirically determined. Increasing the number of units leads to over-fitting (the classification performance of the model evaluated on the validation set is lower than that obtained on the training set). On the other hand, decreasing the number of units degrades the overall classification performance. The activation functions for the state and the gate of biLSTM units were selected to be hyperbolic tangent and sigmoid, respectively. To further reduce the over-fitting, we employed a drop-out layer after the biLSTM layer, which employs regularization by switching off the units with a uniform probability of 0.5. The fully connected layer provides a linear mapping of the biLSTM output to 43 classes of damage. The probability distribution of model prediction is then obtained through exponential normalization by a softmax layer. To train the proposed network, we employed the stochastic gradient descend (SGD) strategy along with the adaptive moment estimation (ADAM) optimizer. The size of mini-batches was set to 600 samples. The learning rate was set to 1–*e*3 with no learning rate scheduler applied.

It is important to notice that the classification of the model is conditional on the applied oscillation pattern. This means that in order to detect the damage, a specific pattern of motion is required to be applied periodically. Developing the model that determines the damage from the arbitrary motion is on our agenda for future research.

## 3 Experiment

**FIGURE 3:**
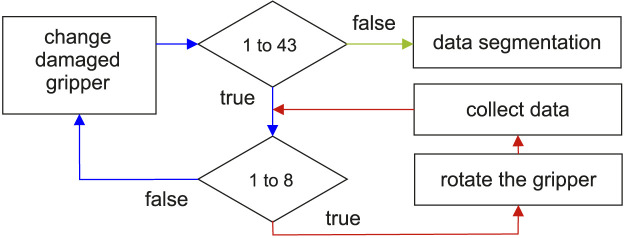
Data collection procedure. The 43 damage combinations are collected by manually replacing the fingers of the Fin Ray gripper (see red line). For each damage combination, the robot collected data for eight roll angles of the gripper. The collected data are further segmented into 125-point-long signals using the sliding window strategy with the 10 points of the shift step between each pair of consecutive segments.

In this section, we present the robotic setup equipped with the soft Fin Ray gripper that we used to test and evaluate the proposed method for damage detection and localization. We also explain the sample set used for data collection of 43 damage configurations, i.e., partial and complete damage at the inner side of the Fin Ray fingers (see Section 3.1 for detail). Finally, the data processing and preparation of training and testing datasets are described in Section 3.2. The flow diagram of data collection and processing is depicted in Figure 3.

**FIGURE 4:**
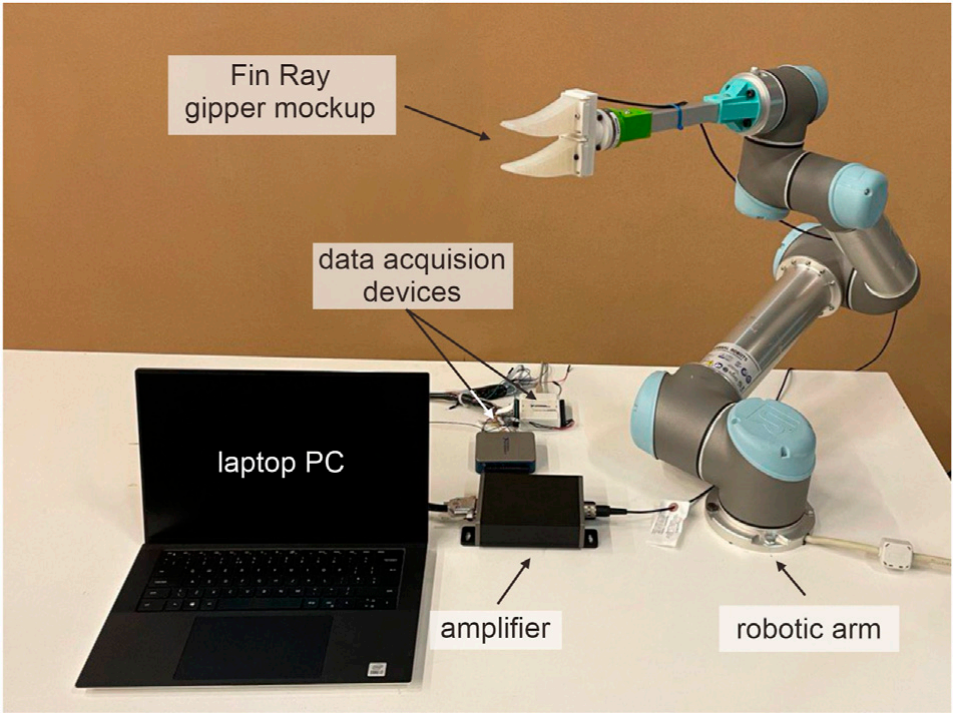
Experimental setup used for data collection.

To evaluate the proposed model, we built an experimental setup, as depicted in Figure 4. To oscillate the object and capture changes in gripper dynamics, we utilized the robotic manipulator (UR5, Universal Robots; Odense, Denmark). The robotic device was operated in a servoing mode with a 300 gain and a 0.1 look-ahead time. These parameters allow the stable operation of the robot to shake the gripper with 22 and 4 Hz excitation magnitude and frequency (see Section 3.2 for further detail). We manufactured the mock-up of a Fin Ray gripper with replaceable silicone fingers. The fingers were cased in a 3D-printed mold using a two-compound silicone (Dragon Skin 20, Smooth-On Inc.; Macungie, Pennsylvania, United States). To capture changes in gripper dynamics, we utilized a six-degree-of-freedom force/torque sensor (Nano43, ATI Technologies; Markham, Ontario, Canada). The force/torque sensor is connected to two data acquisition devices (USD-6002 and USB-6008, National Instruments; Austin, Texas, United States) through a signal amplifier. The force/torque signals were obtained at the sampling frequency of 125 Hz. It is to be noted that we use raw signals obtained directly from strain gauges of the force/torque sensor with no conversion to physical units. The conversion, i.e., linear scaling using a calibration matrix, is not required in our setup as we normalize signals to zero mean and unit variance before training.

The robotic manipulator was controlled in a servoing mode with an update frequency of 125 Hz. The initial configuration of the gripper was aligned to horizontal orientation, as can be observed in Figure 6. To oscillate the gripper, we modulated the angular displacement (pitch) of the last link of the manipulator. The roll of the gripper can also be adjusted before shaking it. In the current experiment, we collected the data on eight levels of roll with a 45° interval. It is to be noted that the last link was extended by an aluminum profile to increase the amplitude of oscillations. This allows using the setup in a laboratory environment with the robotic arm mounted on a table. In a grounded configuration, typically used in industry, the longer link (e.g., second to last) can be oscillated to increase the magnitude of vibrations.

### 3.1 Damage samples

**FIGURE 5:**
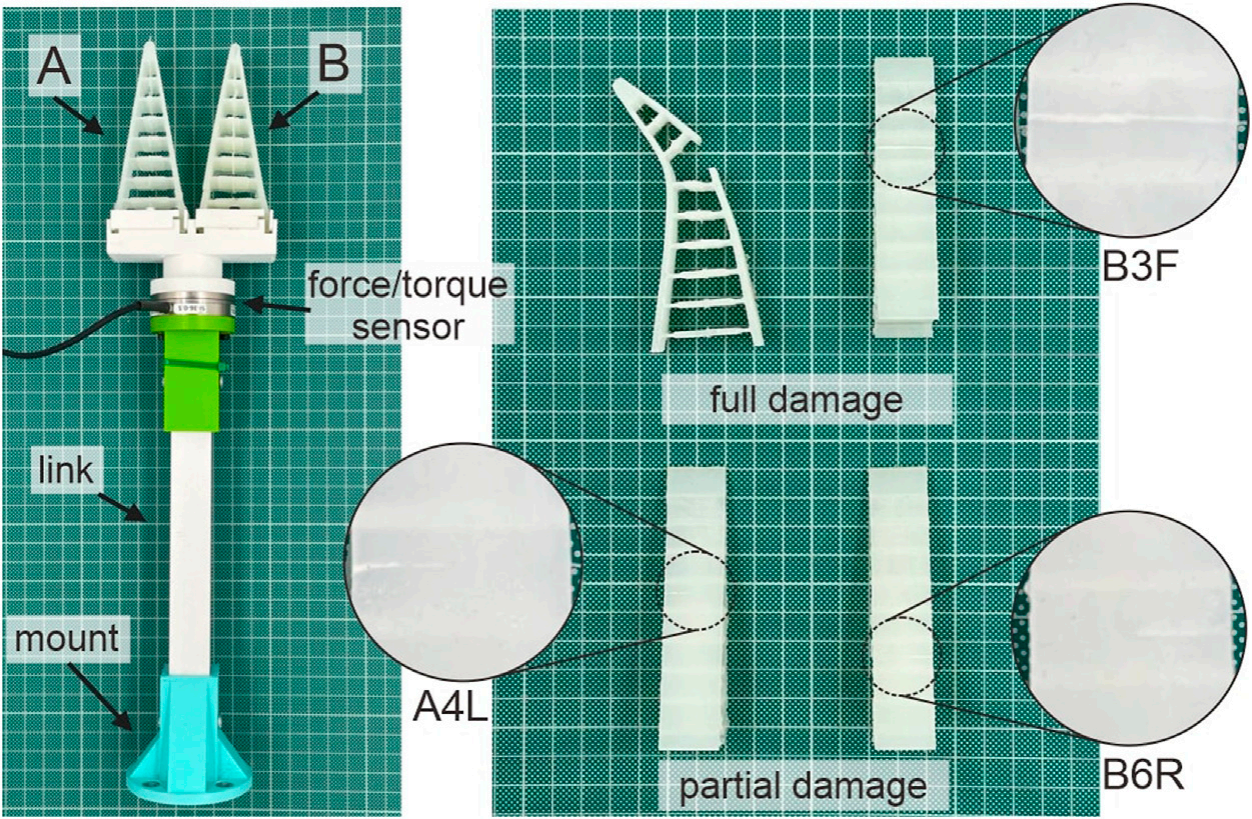
Two-fingered Fin Ray gripper and examples of possible damage. On the left, the gripper equipped with a force/torque sensor at the mounting point of soft fingers is illustrated. Examples of partial and full damage are presented to the right. Partial damage starting from both edges is considered in this study individually.

To test the proposed concept, we prepared a mock-up of the two-fingered Fin Ray gripper. Each finger of the gripper can be changed to the damaged one. Three types of damage considerably affecting the performance of the gripper were introduced, i.e., a complete cut on a single side of the gripper and partial cuts on both edges of the finger (see Figure 5 for example). Taking into account that the damage typically happens at the contact areas of the gripper with objects, we consider only the inner sides of both fingers in this study. For convenience, we labeled damage using the following strategy: we named each finger of the gripper using capital letters of the English alphabet, i.e., A** and B**, for the case of the two-fingered Fin Ray gripper. Then, starting from the top end of the finger, we numbered the area between each cross-beam from 0 to 7, for e.g., A1*. Finally, we labeled three types of damage using the abbreviation F for complete damage and L and R indicating partial damage starting from the left and right edges, respectively. For instance, B3F means that the finger B is damaged across the complete contact area 3. Likewise, A4L indicates partial damage of the contact area 4 starting from its left edge. In total, there are 42 possible combinations of the damage for the current gripper. Additionally, we consider the case with no damage to the gripper, which we denote using label 0. Therefore, we can classify the state of the deformation of the current gripper into 43 classes.

It is important to note that we selected passive soft grippers in our experiment to eliminate the additional factor of actuation. The interaction of the damaged gripper with a third-party object is beyond the scope of the current study. In future works, the actuation can be incorporated as an additional input signal, for instance, or even used as the main source of actuation to determine the changes in dynamics. Additionally, the performance of the gripper with various damage sites can be evaluated during interaction with arbitrary objects. The damage types, in this case, can be further classified as severe damage (the gripper is no longer applicable and requires human attention) and damage that allows particular interactions. In a similar fashion, this approach can be scaled-up to multiple fingers and fingers with different topologies.

### 3.2 Dataset

**FIGURE 6:**
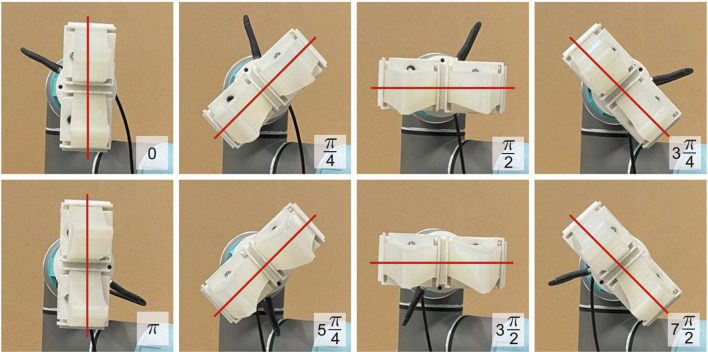
Eight roll angles of the gripper used for data collection. The angular displacement between each neighboring pair orientation is 45°.

**FIGURE 7:**
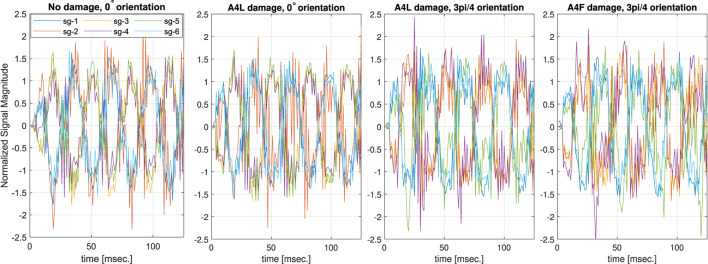
Normalized force (sg-1–sg-3) and torque (sg-4–sg-6) strain gauge signals for four damage configurations. The plots show that differences between two signals of different damage locations but in the same gripper orientation are not obvious.

We collected two sets of data for each damage configuration. The first set of data was used for training and cross-validation and consisted of 25 recording trials. The second set of five recording trials was collected for testing for the corresponding damage configuration but from different Fin Ray fingers, which we additionally prepared to evaluate the generalization of the learning approach. In each data collection trial, we shook the gripper with an angular magnitude of *π*/22 and frequency of 4 Hz for eight orientations of the gripper (see Figure 6). The combination of the magnitude and frequency was selected based on the device’s characteristics. Further increase in frequency or magnitude leads to skipping the motion steps or jerky movement of the robotic arm. The recording time for each orientation was 5 s. Thus the total number of recordings for 43 classes was 8,600 (43 × 8 × 25) for training and 1,720 (43 × 8 × 5) for testing sets. Each recording was further segmented into short time-series data using a sliding window of 125 points length with 10 points of the shift step. Examples of time-series segments for different deformations and gripper orientations are illustrated in Figure 7. As shown in Figure 7, the dominant frequency of signals corresponds to 4 Hz, i.e., the signal of applied excitation. The signal, however, consists of additional frequency components (as the reviewer has mentioned) that play an utmost important role in our approach. This additional frequency captures morphological and material aspects of the gripper, as well as possible damage. This means that the introduced damage affects the frequency content of respective magnitudes to the ones captured from the non-damaged gripper. The segmentation strategy that we applied allows the classifier to be invariant to the initial condition and improve the generalization of the model. The total size after segmentation of the training and testing datasets becomes 54,825 and 10,965 six-dimensional signals, respectively. It is to be noted that we did not apply any filter to the data to prevent loss of important information. Additionally, the natural noise in data prevents over-fitting of the network, which further improves its generalization.

## 4 Results and discussion

The goal of the current section is to evaluate the proposed classification approach in terms of damage detection and localization. Damage detection is the ability of the model to determine the presence of any damage at the current configuration of the gripper. Damage localization is, on the other hand, the classification accuracy of a particular damage type and location (see Section 4.1 for detail). Additionally, in Section 4.2, we analyze the effect of the input space design in terms of the dimension of applied actions, i.e., the orientation of the gripper, and the number of data points of the time series.

### 4.1 Damage detection and localization

**FIGURE 8:**
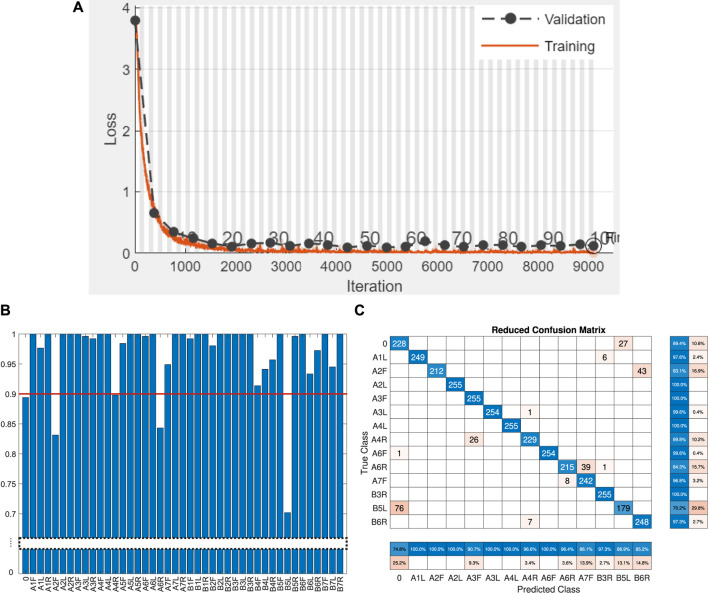
**(A)** Training progress of the biLSTM network. The validation is performed using unseen data during training, which we also use for testing (see Section 3.2). Damage localization results. **(B)** Classification results for 43 individual damage configurations. The red line indicates the threshold used to reduce the number of classes in the confusion matrix in **(C)**. **(C)** Reduce confusion matrix. Only damage configurations with classification accuracy up to 90% are presented to increase readability. The original 43 × 43 confusion matrix is sparse as much damage exhibits a near-perfect classification.

To evaluate the detection and localization of damage on the two-fingered Fin Ray gripper, we trained the model (the loss function of training and validation is shown in Figure 8A) and tested it using the datasets from Section 3.2. The overall classification result for unseen testing data was over 97%. To reveal the damage detection rate, i.e., accuracy for the “no-damage” class, we plot the classification results for all test classes in Figure 8B. The accuracy of the correct “no-damage” prediction was 89.4%. If we consider the binary case of detecting if there is damage or not, then the damage detection rate is over 99%. Both precision and recall, in this case, are over 0.99.

For further analysis, we plotted the confusion matrix in Figure 8C for classes where the classification accuracy was below 90% (the threshold is represented by a red line in Figure 8B). This helps to reduce the number of entries in the matrix and see the most misclassified test-prediction pairs. The classifier tends to confuse the no-damage class with the B5L one. This also holds the other way around, where the B5L was misclassified as a “no-damage” case in 27 out of 255 tests. It is reasonable to consider that the dataset of B5L with an overall classification accuracy of 70% is rather an outlier as the result for A5L (symmetric damage in the other finger of a gripper) is nearly perfect with 98% detection accuracy (see Figure 8B). The accuracy for 5BL is a bit lower than that of others, presumably due to sticking of the cut edges (of the damage). Therefore, some data were detected as “no-damage” for 5BL. We observed this behavior during preliminary experiments. This problem could be potentially resolved by applying silicon oil at the damaged location. However, to keep the experiment more realistic, we decided to leave the samples damaged more naturally. The worst accuracy of 83.1% for localizing full damage belongs to the A2F case. Localizing damage closer to the tip of the gripper is generally more challenging due to lower inertia of the remaining body parts after the damage location.

We also performed the 10-fold cross-validation only for the training dataset. Each test result from cross-validation was concatenated, and the final classification accuracy was computed. The overall accuracy was 99%. The worst performance was observed for the A6R case with 93%, whereas the rest of the damage cases are correctly classified as 97% or higher. The reason for that is the segmentation strategy used in Section 3.2, where the signals overlap one another. Therefore, part of the signal used in training can appear as a part of testing, which simplifies the task to the classifier. This cross-validation analysis once again justifies the choice of using the unseen data for testing that we utilized in the main experiment.

### 4.2 Input dimension analysis

**FIGURE 9:**
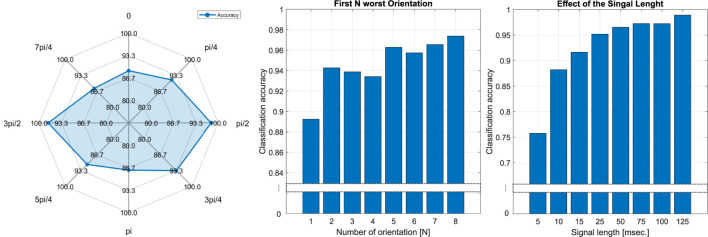
Damage classification results. Left, classification accuracy for different orientations of the gripper illustrated in Figure 6. Middle, the damage localization accuracy for combinations of N worst gripper orientations (ranked according to their classification accuracy). Right, the classification results with respect to the number of data points in the time series.

To analyze the effect of each roll angle that we used in the action dimension of the model, we ran the training for reduced input size of the individual gripper orientation. The results of this experiment are presented in Figure 9. The classification accuracy significantly differs for various roll angles. The classification results for various angles are different mainly due to the morphology of the gripper, i.e., two fingers are vertically aligned. The changes in the dynamics while shaking the gripper in certain directions are more prominent than other changes. This mainly explains the reason for the noticeable difference in classification accuracy. The best performance is observed for *π*/2 and 3*π*/2 (horizontal configuration, i.e., orthogonal to the direction of oscillation) having nearly 96% classification accuracy. This means that even the data collected for a single gripper orientation can be already used for damage detection. To perform further analysis, we sorted roll angles accordingly to classification accuracy and ran the training for input spaces with first N orientations having the worst solo performance (see the middle plot in Figure 9). It is clearly seen that 2–4 orientations having 94% detection are worse than either *π*/2 or 3*π*/2 angles only. Furthermore, the accuracy for *π*/2 and 3*π*/2 orientations is as high as that of the other roll angles combined. To analyze the effect of the number of samples in the sequence, we used all eight orientations and ran training for eight different lengths of signals (see the right plot in Figure 9). Five data points of the 48-dimensional signal (6 strain signals × 8 roll angles) can produce a detection rate of over 75%. The signal length of 10 samples, which is almost a half period of applied oscillation, exhibits a classification accuracy of roughly 87%. The detection rate increases over 95% for the sequence equal to the full period of a sine wave applied to oscillate the gripper. It is important to notice that the initial condition of each signal in the training and testing datasets is shifted according to the segmentation rule presented in Section 3.2. This means that any 25 points of the signal can produce a reasonable level of damage localization. Approximately 75 data points are enough to realize the full potential of the classification model.

## 5 Conclusion and future work

In this paper, we develop a novel approach to damage detection and localization on a soft Fin Ray gripper. This identifies damage based on changes in dynamics observed at the mounting point of the gripper, which in turn does not require embedding strain sensors into the gripper and visual tracking of morphological changes. The proposed approach classifies the location of both partial and full cuts on either of the two fingers of the Fin Ray gripper. The proposed approach is evaluated using the unseen data with a damage detection accuracy of 99% and average localization of the damage of 97%. We have also shown that oscillating the gripper with an optimal orientation can outperform the classification accuracy of the model with an input of six roll angles combined. Additionally, we discovered that the length of the signal of the order of complete sine wave of oscillation can provide a reasonable level of damage localization.

There are several research directions that we are planning to explore in our future work. First, we would like to develop a model that identifies damage locations based on arbitrary movement, with no need to stop the operation of the robot and perform a predefined pattern of oscillatory movement. Second, the regression model can be beneficial to damage detection and localization of a continuum of the gripper’s matter. Third, it would be great to explore non-invasive material identification and characterization using non-linear dynamics. Finally, we would like to extend our approach to other types of the gripper with additional actuation methods, such as tendon-driven, pneumatics, and hydraulics.

## Data Availability

The raw data supporting the conclusion of this article will be made available by the authors, without undue reservation.

## References

[B1] Abazarsa F. Nateghi F. Ghahari S. Taciroglu E. (2016). Extended blind modal identification technique for nonstationary excitations and its verification and validation. J. Eng. Mech. 142, 04015078. 10.1061/(asce)em.1943-7889.0000990

[B2] Avci O. Abdeljaber O. Kiranyaz S. Hussein M. Gabbouj M. Inman D. J. (2021). A review of vibration-based damage detection in civil structures: From traditional methods to machine learning and deep learning applications. Mech. Syst. signal Process. 147, 107077. 10.1016/j.ymssp.2020.107077

[B3] Avci O. Abdeljaber O. (2016). Self-organizing maps for structural damage detection: A novel unsupervised vibration-based algorithm. J. Perform. Constr. Facil. 30, 04015043. 10.1061/(asce)cf.1943-5509.0000801

[B4] Ay A. M. Wang Y. (2014). Structural damage identification based on self-fitting armax model and multi-sensor data fusion. Struct. Health Monit. 13, 445–460. 10.1177/1475921714542891

[B5] Carden E. P. Brownjohn J. M. (2008). Arma modelled time-series classification for structural health monitoring of civil infrastructure. Mech. Syst. signal Process. 22, 295–314. 10.1016/j.ymssp.2007.07.003

[B6] Chen F. Xu W. Zhang H. Wang Y. Cao J. Wang M. Y. (2018). Topology optimized design, fabrication, and characterization of a soft cable-driven gripper. IEEE Robot. Autom. Lett. 3, 2463–2470. 10.1109/lra.2018.2800115

[B7] Choe D.-E. Kim H.-C. Kim M.-H. (2021). Sequence-based modeling of deep learning with lstm and gru networks for structural damage detection of floating offshore wind turbine blades. Renew. Energy 174, 218–235. 10.1016/j.renene.2021.04.025

[B8] Cunha Á. Caetano E. (2006). Experimental modal analysis of civil engineering structures. Sound Vib. 40 (6).

[B9] George Thuruthel T. Bosman A. W. Hughes J. Iida F. (2021). Soft self-healing fluidic tactile sensors with damage detection and localization abilities. Sensors 21, 8284. 10.3390/s21248284 34960380 PMC8706411

[B10] George Thuruthel T. Gardner P. Iida F. (2022). Closing the control loop with time-variant embedded soft sensors and recurrent neural networks. Soft Robot 9, 1–10. 10.1089/soro.2021.0012 35446168 PMC9805858

[B11] Georgopoulou A. Bosman A. W. Brancart J. Vanderborght B. Clemens F. (2021). Supramolecular self-healing sensor fiber composites for damage detection in piezoresistive electronic skin for soft robots. Polymers 13, 2983. 10.3390/polym13172983 34503023 PMC8433753

[B12] Ghahari S. Abazarsa F. Taciroglu E. (2017). Blind modal identification of non-classically damped structures under non-stationary excitations. Struct. Control Health Monit. 24, e1925. 10.1002/stc.1925

[B13] Goi Y. Kim C.-W. (2017). Damage detection of a truss bridge utilizing a damage indicator from multivariate autoregressive model. J. Civ. Struct. Health Monit. 7, 153–162. 10.1007/s13349-017-0222-y

[B14] Graves A. Liwicki M. Fernández S. Bertolami R. Bunke H. Schmidhuber J. (2008). A novel connectionist system for unconstrained handwriting recognition. IEEE Trans. Pattern Anal. Mach. Intell. 31, 855–868. 10.1109/tpami.2008.137 19299860

[B15] Graves A. Schmidhuber J. (2005). Framewise phoneme classification with bidirectional lstm and other neural network architectures. Neural Netw. 18, 602–610. 10.1016/j.neunet.2005.06.042 16112549

[B16] Gul M. Catbas F. N. (2008). Ambient vibration data analysis for structural identification and global condition assessment. J. Eng. Mech. 134, 650–662. 10.1061/(asce)0733-9399(2008)134:8(650)

[B17] Gul M. Catbas F. N. (2011). Damage assessment with ambient vibration data using a novel time series analysis methodology. J. Struct. Eng. (N. Y. N. Y). 137, 1518–1526. 10.1061/(asce)st.1943-541x.0000366

[B18] Heindel L. Hantschke P. Kästner M. (2022). A data-driven approach for approximating non-linear dynamic systems using lstm networks. Procedia Struct. Integr. 38, 159–167. 10.1016/j.prostr.2022.03.017

[B19] Hochreiter S. Schmidhuber J. (1997). Long short-term memory. Neural Comput. 9, 1735–1780. 10.1162/neco.1997.9.8.1735 9377276

[B20] Hong Y. Su M. (2012). Multifunctional self-healing and self-reporting polymer composite with integrated conductive microwire networks. ACS Appl. Mat. Interfaces 4, 3759–3764. 10.1021/am3009746 22747085

[B21] Hughes J. Culha U. Giardina F. Guenther F. Rosendo A. Iida F. (2016). Soft manipulators and grippers: A review. Front. Robot. AI 3, 69. 10.3389/frobt.2016.00069

[B22] Khatib M. Zohar O. Saliba W. Haick H. (2020). A multifunctional electronic skin empowered with damage mapping and autonomic acceleration of self-healing in designated locations. Adv. Mat. 32, 2000246. 10.1002/adma.202000246 32173928

[B23] Khodabandehlou H. Pekcan G. Fadali M. S. (2019). Vibration-based structural condition assessment using convolution neural networks. Struct. Control Health Monit. 26, e2308. 10.1002/stc.2308

[B24] Kingsley D. A. Quinn R. D. (2002). “Fatigue life and frequency response of braided pneumatic actuators,” in Proceedings 2002 IEEE International Conference on Robotics and Automation (Cat. No. 02CH37292) (IEEE), 2830–2835.

[B25] Krishnan Nair K. Kiremidjian A. S. (2007). Tmie series based structural damage detection algorithm using Gaussian mixtures modeling. J. Dyn. Syst. Meas. Control 129 (3). 10.1115/1.2718241

[B26] Li F. Gao S. Lu Y. Asghar W. Cao J. Hu C. (2021). Multi‐mode pain‐perceptual system: Bio‐inspired multi‐mode pain‐perceptual system (MMPPS) with noxious stimuli warning, damage localization, and enhanced damage protection (adv. Sci. 10/2021). Adv. Sci. 8, 2170055. 10.1002/advs.202170055 PMC8132158 34026450

[B27] Li X. Zhang W. (2022). Physics-informed deep learning model in wind turbine response prediction. Renew. Energy 185, 932–944. 10.1016/j.renene.2021.12.058

[B28] Markvicka E. J. Tutika R. Bartlett M. D. Majidi C. (2019). Soft electronic skin for multi-site damage detection and localization. Adv. Funct. Mat. 29, 1900160. 10.1002/adfm.201900160

[B29] Mayer H. Gomez F. Wierstra D. Nagy I. Knoll A. Schmidhuber J. (2008). A system for robotic heart surgery that learns to tie knots using recurrent neural networks. Adv. Robot. 22, 1521–1537. 10.1163/156855308x360604

[B30] Mosadegh B. Polygerinos P. Keplinger C. Wennstedt S. Shepherd R. F. Gupta U. (2014). Pneumatic networks for soft robotics that actuate rapidly. Adv. Funct. Mat. 24, 2163–2170. 10.1002/adfm.201303288

[B31] Padil K. H. Bakhary N. Abdulkareem M. Li J. Hao H. (2020). Non-probabilistic method to consider uncertainties in frequency response function for vibration-based damage detection using artificial neural network. J. Sound Vib. 467, 115069. 10.1016/j.jsv.2019.115069

[B32] Pu W. Fu D. Wang Z. Gan X. Lu X. Yang L. (2018). Realizing crack diagnosing and self-healing by electricity with a dynamic crosslinked flexible polyurethane composite. Adv. Sci. (Weinh). 5, 1800101. 10.1002/advs.201800101 29876226 PMC5978978

[B33] Salawu O. (1997). Detection of structural damage through changes in frequency: A review. Eng. Struct. 19, 718–723. 10.1016/s0141-0296(96)00149-6

[B34] Terryn S. Langenbach J. Roels E. Brancart J. Bakkali-Hassani C. Poutrel Q.-A. (2021). A review on self-healing polymers for soft robotics. Mater. Today 47, 187–205. 10.1016/j.mattod.2021.01.009

[B35] Thostenson E. T. Chou T.-W. (2006). Carbon nanotube networks: Sensing of distributed strain and damage for life prediction and self healing. Adv. Mat. 18, 2837–2841. 10.1002/adma.200600977

[B36] Zia T. Zahid U. (2019). Long short-term memory recurrent neural network architectures for Urdu acoustic modeling. Int. J. Speech Technol. 22, 21–30. 10.1007/s10772-018-09573-7

